# If these myocytes could talk, they would speak the language of metabolites

**DOI:** 10.1172/JCI156296

**Published:** 2022-01-18

**Authors:** Logan R.J. Bailey, Jennifer Davis

**Affiliations:** 1 Molecular and Cellular Biology Graduate Program,; 2 Medical Scientist Training Program,; 3 Department of Bioengineering,; 4 Department of Lab Medicine and Pathology,; 5 Institute for Stem Cell and Regenerative Medicine, and; 6 Center for Cardiovascular Biology, University of Washington, Seattle, Washington, USA.

## Abstract

Cardiac wound healing following ischemic injury requires a well-described spatiotemporal progression of events involving multiple cell types and cell-cell interactions. While cellular crosstalk among immune cell, endothelial cell, and fibroblast populations is known to regulate these progressive phases, the role of cardiac myocytes in controlling the wound-healing program is unclear. In this issue of the *JCI*, Li et al. describe a mechanism of cellular crosstalk between cardiac myocytes and fibroblasts that disrupts nonmyocyte cell function and worsens wound healing outcomes following myocardial infarction (MI). This tour de force study used an arsenal of multidisciplinary approaches to identify a central role for the ectonucleotidase ENPP1 in this process. These findings have clear therapeutic implications, as the authors identified a small molecular inhibitor of ENPP1 that improved post-MI outcomes in mice. These exciting data provide impactful mechanistic information that advance the field’s understanding of cardiac repair and remodeling.

## Cellular crosstalk in cardiac wound healing

Ischemic heart disease is a leading cause of heart failure and death worldwide. Following myocardial infarction (MI), cardiac wound healing progresses in a well-described series of steps involving multiple cell types. Immediately following injury, extracellular ATP release by necrotic cardiomyocytes recruits a polymorphonuclear cell infiltrate to the heart to clear necrotic cell debris (1). Macrophages then replace the infiltrate and, together with cardiac fibroblasts and endothelial cells, orchestrate the formation of granulation tissue and ultimately a permanent collagenous scar (1). Cellular crosstalk within the healing heart is an integral part of the wound-healing process, and many unidirectional mechanisms of crosstalk have been elucidated (2, 3). Much of what we know is immune centric: invading and tissue-resident immune cells secrete numerous cytokines, which modulate the behavior of nonleukocyte populations (4). Among other cell populations, endothelial cells help recruit immune cells to the heart, control tissue oxygenation, and secrete paracrine-acting growth factors (5). Cardiac fibroblasts largely determine the heart’s microenvironment and physical crosstalk between cells (6, 7), and recent single-cell studies have revealed that fibroblasts are hubs for outgoing signaling molecules in the heart (8). Though cardiac myocytes make up most of the heart volume, limited cell-cell communication roles have been described for these cells. However, cardiomyocytes are known to play a role in activating the innate immune system and in promoting neoangiogenesis (4, 5). Due to the changing cellular composition of the heart and the complex interplay between different cell types following MI, elucidating mechanisms of cellular crosstalk is critical to understanding cardiac wound healing.

Attempts to therapeutically target cellular crosstalk in cardiac wound healing have been frustrated by the double-edged sword aspect of crosstalk mechanisms: most theoretically actionable cell-cell communication mechanisms are heterogenous, having both beneficial and deleterious consequences, depending on when they occur and how long they persist. While most of these proposed therapies are immunomodulatory and show promise in animal models, they fail to translate benefit to human patients (9). As clinicians have few therapeutic tools to improve post-MI heart function, a better understanding of cellular crosstalk in cardiac wound healing is necessary for the creation of next-generation therapeutics for ischemic heart disease.

## Cardiac fibroblast ENPP1 metabolizes extracellular ATP

Extracellular release of ATP, a damage-associated molecular pattern (DAMP), from necrotic cardiomyocytes initiates the wound-healing process following MI (1). Accumulation of extracellular ATP is limited by membrane-bound ectonucleotidases on invading immune cells and tissue-resident cells, which hydrolyze ATP, providing an antiinflammatory effect and modulating local purinergic signaling (10, 11). In this issue of the *JCI*, Li et al. hypothesized that metabolism of extracellular ATP by tissue ectonucleotidases could regulate intercellular communication between myocytes and nonmyocytes in the infarcted region (12). Here, using ENPP1 mutant mice, several fibroblast lineage-tracing mouse models, single-cell RNA sequencing, and enzymatic assays, Li et al. convincingly demonstrate that (a) ENPP1 is the principle ectonucleotidase that degrades extracellular ATP in the heart and (b) cardiac fibroblasts are the primary cell type expressing ENPP1 in the injured heart. Targeted deletion of ENPP1 in cardiac fibroblasts following MI rescued several parameters of adverse cardiac remodeling, and single-cell RNA sequencing revealed downregulation of proinflammatory, apoptotic, and fibrotic pathways in hearts with cardiac fibroblast deletion of ENPP1. Whereas previous studies had focused on immune cell ectonucleotidases in regulating post-MI wound healing (13–15), Li et al. demonstrate that cardiac fibroblast ENPP1 is the major regulator of extracellular ATP metabolism in the postinfarct heart and that cardiac fibroblast ENPP1-dependent signaling substantially affects post-MI remodeling (12).

## Disrupting nonmyocyte pyrimidine synthesis to regulate heart repair

**Figure 1:**
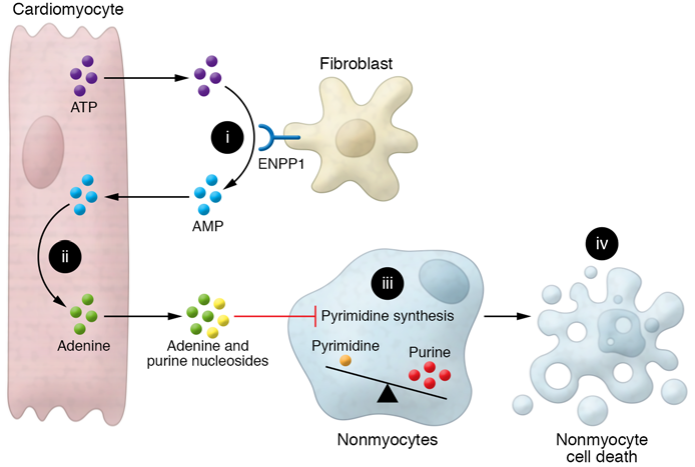
A metabolic conversation between cardiac myocytes and fibroblasts disrupts nonmyocyte cell function in post-MI wound healing. (i) Cardiomyocyte-derived extracellular ATP is metabolized to AMP by cardiac fibroblast ENPP1. (ii) ENPP1-derived AMP is metabolized to adenine within cardiomyocytes and released into the extracellular space. (iii) Cardiomyocyte-derived adenine and other purine nucleosides inhibit pyrimidine synthesis in nonmyocyte cells. (iv) Purine-pyrimidine imbalance in nonmyocyte cells leads to genotoxic stress, cell death, and poor wound-healing outcomes.

Following up on this finding, Li et al. performed a series of elegant coculture experiments showing that ENPP1 expression in fibroblasts induced cardiomyocytes to secrete proapoptotic molecules that selectively target nonmyocyte populations. Mass spectrometry analysis and further coculture experiments revealed that hydrolysis of ATP to AMP by ENPP1 was required for this cell-death response (Figure 1). AMP generated by ENPP1 was further metabolized to the nucleobase adenine within cardiomyocytes (Figure 1), and release of cardiomyocyte-derived adenine in combination with other purine nucleosides inhibited pyrimidine synthesis at the UMP synthase step in nonmyocyte populations (Figure 1). This inhibition of pyrimidine biosynthesis resulted in an imbalance of purine and pyrimidine nucleotides in nonmyocytes, leading to genotoxic stress, activation of p53-mediated cell death, and poor wound-healing outcomes (Figure 1 and ref. 12). Ultimately, the promotion of cell death specifically in nonmyocytes was likely related to cell proliferation: while intrinsically nonproliferative cardiomyocytes were never affected by these apoptotic signals, cell-cycle inhibition in nonmyocytes rescued the cell death effect of conditioned media. However, more work remains to be done to confirm that this signaling pathway does not affect myocytes, as myocytes should require a balanced nucleotide pool both for DNA damage repair and for increased polyploidization occurring during compensatory hypertrophy after MI. Regardless, the metabolite conversation between cardiac myocytes and fibroblasts described in Li et al. (12) is an important paradigm of cell-cell communication with profound ramifications for cardiac wound healing. Notably, in contrast to other previously described mechanisms of cellular crosstalk, this metabolic assault by cardiomyocytes on nonmyocyte cell types seems uniquely counterproductive and presents a compelling opportunity for pharmacological targeting.

## Maladaptive ATP/ENPP1 metabolite signaling is pharmacologically actionable

Following characterization of this ENPP1 pathway, Li et al. screened a large library of compounds to identify small molecule inhibitors of ENPP1. This screen identified myricetin, a polyphenolic flavonoid, which potently inhibited ENPP1 activity. Intraperitoneal administration of myricetin improved outcomes in a murine model of MI, where mice receiving myricetin had improved systolic function, decreased chamber remodeling, reduced scar size, and increased capillary density. This functional rescue appears to be due to myricetin’s ENPP1 inhibitory function, as myricetin rescued pyrimidine biosynthesis and the DNA damage response in nonmyocyte cells. Finally, Li et al. performed a metabolomic analysis of serum from control and myricetin-treated animals and showed that serum orotidine levels could be used to monitor wound healing via this process in the infarcted heart (12). These applied findings present a promising drug target for treating ischemia-induced cardiac remodeling, laying the groundwork for potential clinical application of ENPP1 inhibitors. Remarkably, ENPP1 inhibitors could be a first-in-class therapeutic targeting cell-cell crosstalk to promote beneficial remodeling after MI.

## Conclusion and outlook

Here, the authors identify a cardiomyocyte-dependent paradigm of cellular crosstalk governing post-MI remodeling and show that this pathway is clinically actionable. In testing their ENPP1 inhibitor, Li et al. used a clinically relevant time course of drug administration, with myricetin being delivered after injury (12). This preclinical study provides excellent motivation for the advancement of ENPP1 inhibitor therapy, as treatment could easily be started as early as hours after MI and continued for the appropriate period in human patients. One point of caution is that we do not yet fully understand the temporal dynamics and roles of cardiac ENPP1 expression following MI from this study (12), and more work will be needed to determine the optimal therapeutic time window for treatment. It would be reasonable to predict that early treatment with pan-ENPP1 inhibitors could increase the inflammatory response, and early (before 7 days after MI) inflammatory dynamics were not explored in this study following myricetin treatment. It appears that fibroblast ENPP1 increased by 3 days after MI, peaking by day 7. Beneficial, therapeutic effects would likely be driven by inhibition occurring in this time window. Furthermore, Li et al. proposed a serum biomarker, orotidine, whose levels rose following ENPP1 inhibition, reflecting rescued nonmyocyte pyrimidine synthesis that could be serially measured to monitor the functional status of this mechanism (12). This potential biomarker presents a superb opportunity for future study in human patients, both for investigating whether this mechanism occurs in post-MI remodeling in humans and for monitoring the effectiveness of potential treatments targeting this pathway.

An interesting consideration we are still left with is the teleological reason for why this process occurs, as this mechanism seems completely maladaptive for postinfarct wound healing. For example, is ENPP1-mediated signaling a broader mechanism for limiting inflammation in the heart that is beneficial in chronic inflammatory conditions, but detrimental in acute wound-healing processes? This mechanism could have evolved in response to conditions such as chronic myocarditis to beneficially reduce immune-mediated cardiomyocyte injury and myocardial fibrosis. Its role in the acute injury response was unlikely under similar evolutionary pressures, as MI typically occurs at ages after reproduction is completed. It will be informative to further explore this pathway in other modes of cardiac injury and to delineate which circumstances prove beneficial versus maladaptive. Additionally, it will be interesting to explore whether this mechanism contributes to maintaining cardiomyocyte cell-cycle exit in the adult mammalian heart. While Li and coauthors demonstrated that cell-cycle inhibition in nonmyocyte cells allowed these cells to evade cell death, they did not directly investigate cardiomyocyte proliferation, cell death, or DNA synthesis following myricetin treatment in vivo. It is possible that inhibition of this metabolic assault on proliferating cells could also allow for further beneficial remodeling by cardiomyocytes, including by increased proliferation. Ultimately, beyond presenting profoundly interesting and clinically relevant findings, these experiments (12) were exceptionally well constructed and will serve as a blueprint for how cell-cell crosstalk should be rigorously studied. This work will lay the foundation for future work studying not only this specific pathway, but for many studies concerned with how cells communicate with one another. We congratulate Li and colleagues on their excellent study (12), and we look forward to seeing how this story continues to develop.

## Version 1 01/18/2022

Electronic publication
